# Efficiency of Antimicrobial Peptides Against Multidrug-Resistant Staphylococcal Pathogens

**DOI:** 10.3389/fmicb.2022.930629

**Published:** 2022-06-09

**Authors:** Mi Nguyen-Tra Le, Miki Kawada-Matsuo, Hitoshi Komatsuzawa

**Affiliations:** Department of Bacteriology, Graduate School of Biomedical and Health Sciences, Hiroshima University, Hiroshima, Japan

**Keywords:** antimicrobial peptides, staphylococci, MRSA, MRSE, human AMPs, bacteriocins

## Abstract

Antibiotics play a vital role in saving millions of lives from fatal infections; however, the inappropriate use of antibiotics has led to the emergence and propagation of drug resistance worldwide. Multidrug-resistant bacteria represent a significant challenge to treating infections due to the limitation of available antibiotics, necessitating the investigation of alternative treatments for combating these superbugs. Under such circumstances, antimicrobial peptides (AMPs), including human-derived AMPs and bacteria-derived AMPs (so-called bacteriocins), are considered potential therapeutic drugs owing to their high efficacy against infectious bacteria and the poor ability of these microorganisms to develop resistance to them. Several staphylococcal species including *Staphylococcus aureus*, *Staphylococcus epidermidis*, *Staphylococcus haemolyticus*, and *Staphylococcus saprophyticus* are commensal bacteria and known to cause many opportunistic infectious diseases. Methicillin-resistant *Staphylococci*, especially methicillin-resistant *S. aureus* (MRSA), are of particular concern among the critical multidrug-resistant infectious Gram-positive pathogens. Within the past decade, studies have reported promising AMPs that are effective against MRSA and other methicillin-resistant *Staphylococci*. This review discusses the sources and mechanisms of AMPs against staphylococcal species, as well as their potential to become chemotherapies for clinical infections caused by multidrug-resistant staphylococci.

## Introduction

*Staphylococci* are Gram-positive, facultative anaerobe, and some staphylococcal species are commensal bacteria in humans, mainly on the skin. *Staphylococci* are clinically classified into two groups, coagulase-positive *Staphylococcus aureus* and coagulase-negative staphylococci (CoNS). *Staphylococcus aureus* shows higher virulence than CoNS because *S. aureus* produces various virulence factors such as exotoxins, immune evasion factors, adhesins, and exoenzymes (Lowy, 1998; Foster, 2004). *Staphylococcus aureus* is associated with both human commensal and clinical infections (Lowy, 1998; Wertheim et al., 2005). *Staphylococcus aureus* can cause skin and soft tissue disease, pleuropulmonary disease, medical device-related bloodstream infections, food poisoning, and even infective endocarditis or osteomyelitis (Lowy, 1998; Lindsay and Holden, 2004; Tong et al., 2015; Kavanagh et al., 2018; Lakhundi and Zhang, 2018; Oliveira et al., 2018; Turner et al., 2019; Horino and Hori, 2020; Pimentel de Araujo et al., 2021). Although less virulent than *S. aureus*, CoNS including *Staphylococcus epidermidis, Staphylococcus haemolyticus, Staphylococcus saprophyticus, Staphylococcus capitis, Staphylococcus lugdunensis, Staphylococcus hominis, Staphylococcus schleiferi*, and *Staphylococcus warneri* are also important staphylococcal pathogen and are usually associated with hospital infections such as skin and soft tissue disease, sepsis, meningitis, endocarditis, and catheter- or implanted device-mediated infections (Vuong and Otto, 2002; Otto, 2009; Becker et al., 2014; Natsis and Cohen, 2018; Azimi et al., 2020; Parthasarathy et al., 2020).

Since the introduction of methicillin in clinical practice, methicillin-resistant *S. aureus* (MRSA) has evolved by acquisition of *mecA* coding PBP2’ and spread to worldwide (Chambers and DeLeo, 2009). The first report of MRSA was published in 1961 (Barber, 1961). In the 1980s and 1990s, hospital-acquired MRSA strains with multidrug resistance spread across the world (Crossley et al., 1979; Lowy, 1998; Chambers and DeLeo, 2009). Later, community-acquired MRSA strains, which typically cause skin and soft tissue infections in healthy patients, have been firstly reported in the 1980s (Levine et al., 1982; Saravolatz et al., 1982; Lakhundi and Zhang, 2018). In 2004, a livestock-associated MRSA strain was identified from the family of a pig farmer and their pig (Voss et al., 2005; Crespo-Piazuelo and Lawlor, 2021). The increased usage of vancomycin as an alternative to methicillin to treat MRSA infections has led to the emergence of vancomycin-intermediate *S. aureus* (VISA; Hiramatsu et al., 1997) and vancomycin-resistant *S. aureus* (VRSA) strains (Weigel et al., 2003). As with MRSA, methicillin-resistant *S. epidermidis* (MRSE) has also been a serious threat considering its high prevalence in some areas in the world during the 2000s (Carbon, 2000) and its recent global spread (Lee et al., 2018). In addition, the *mecA* gene was also identified in other CoNS including *S. haemolyticus, S. hominis, S. capitis*, and *S. warneri* (Humphries et al., 2020). Furthermore, the reduced susceptibility to glycopeptide has also been reported in *S. epidermidis* and *S. haemolyticus* (Biavasco et al., 2000).

**Figure 1:**
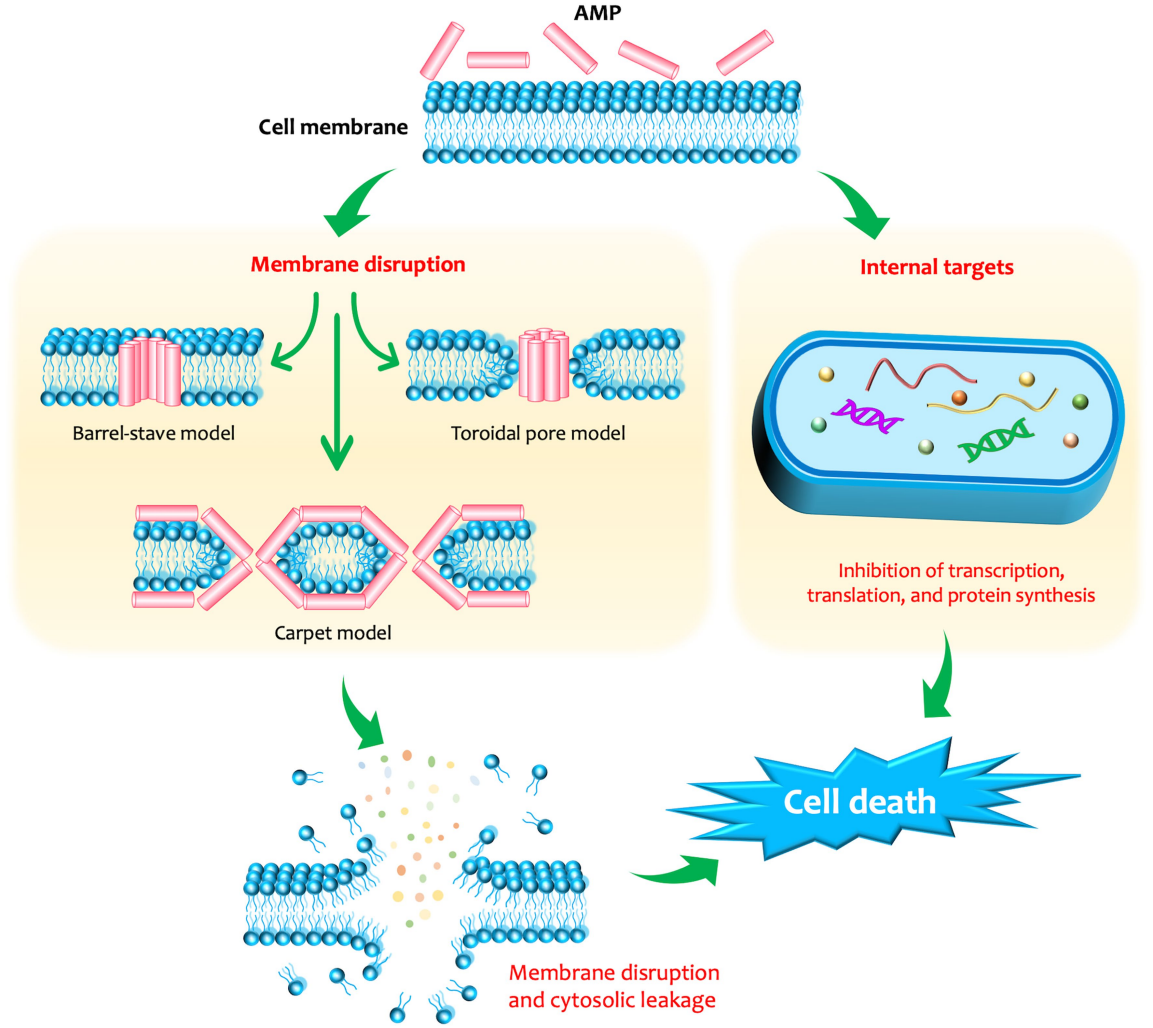
The membrane-disruptive and non-membrane-disruptive antibacterial mechanisms of antimicrobial peptides (AMPs). In the membrane-disruptive mechanisms, three types of interaction can occur between the membrane and the AMPs, including: (i) barrel-stave model: the peptide monomers form a hydrophilic transmembrane channel by arranging parallelly to the phospholipids of the membrane; (ii) carpet model: the peptides solubilize the membrane into micellar structures; and (iii) toroidal model: the lipid moieties fold inward due to the cascade aggregation of peptide monomers, forming a peptide-and-lipid-lined channel.

The ever-increasing burden of global widespread multidrug-resistant bacteria has significantly challenged with the ability of available antibiotics to treat infections and prompted the discovery of novel antimicrobial compounds to overcome the shortage of therapeutic options. Antimicrobial peptides (AMPs) produced by living organisms such as humans and bacteria are candidates for promising strategies to control these superbugs. AMPs generally have a broad spectrum of activity against bacteria, viruses, fungi, and parasites, a specific mode of action, low risk of resistance development, high stability in wide ranges of pH and temperature, low toxicity to eukaryotic cells, and immunomodulatory effects (Zhang and Gallo, 2016). They exhibit antimicrobial activity *via* interaction with bacterial membrane, causing membrane dysfunction and disruption, disturbance of cell wall, DNA/RNA, and protein synthesis (Nguyen et al., 2011; Mahlapuu et al., 2016; Figure 1). Therefore, AMPs represent a novel alternative therapeutic for the control of critical pathogens in the future. However, clinical applications of AMPs raise several concerns such as toxicity, immunogenicity, and hemolytic activity (Moravej et al., 2018; Lei et al., 2019). As mentioned above, *S. aureus* and CoNS have developed resistance to many antimicrobial agents, especially methicillin, and many staphylococcal strains may be resistant to glycopeptides in the future. Therefore, the development of AMPs as new alternative antimicrobial agents against *Staphylococci* is of great importance. In this review, we provide an overview of human and bacterial AMPs that are effective against staphylococcal pathogens, their structures and mode of action, the current stage of investigation, and their potential as therapeutic agents in clinical treatment against staphylococcal infections.

## Human AMPs That Have Antibacterial Activity Against Staphylococcal Pathogens

### Classification of Human AMPs

**Figure 2:**
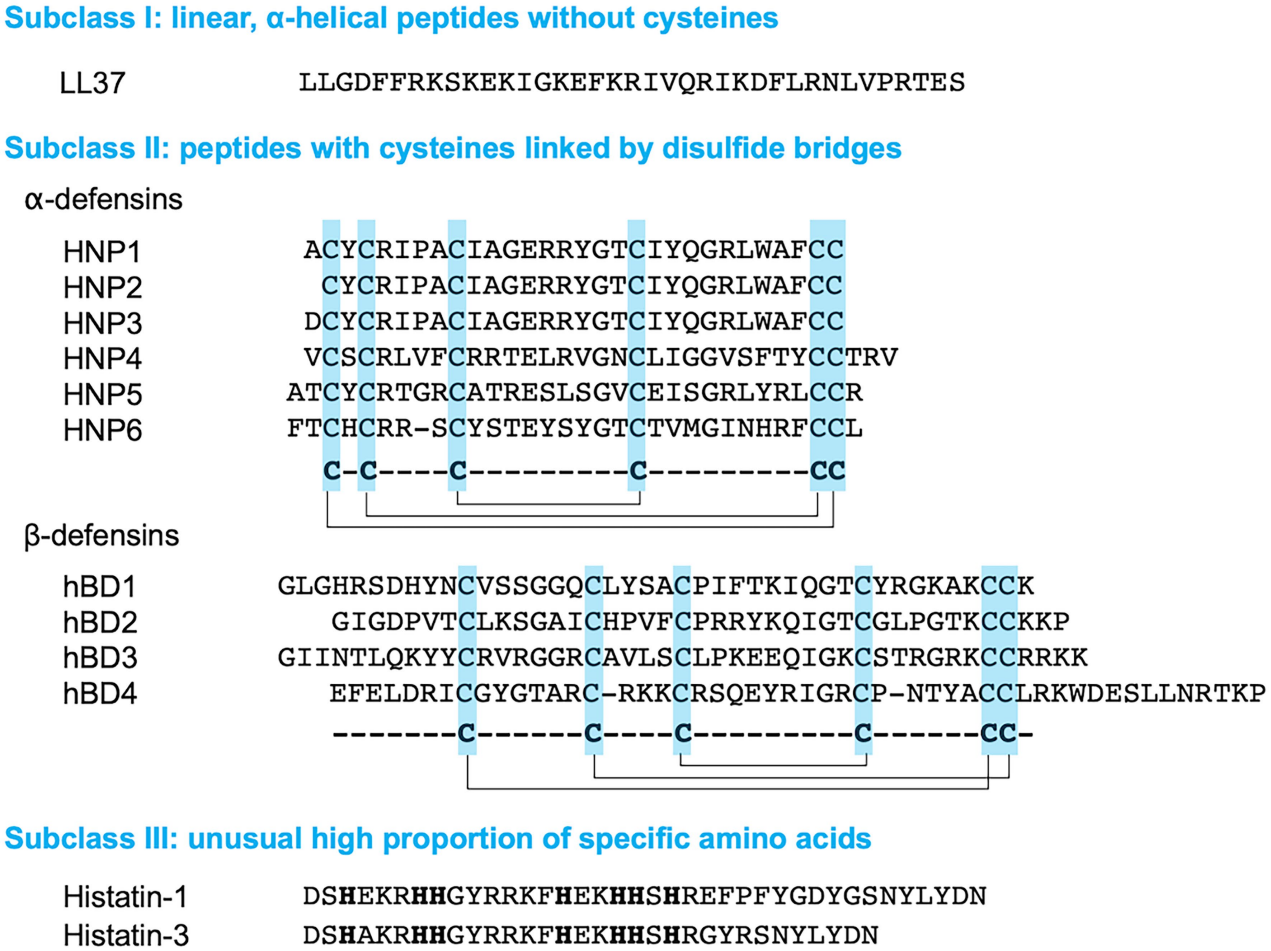
Amino acid sequences of human antimicrobial peptides (AMPs). The disulfide bonds in α- and β-defensins are indicated by solid lines. The histamine residues in Histatin-1 and -3 are indicated by bold letters.

There are various ways to classify AMPs, for example, based on their sequences, structures, or mode of action. Based on structure, AMPs can be classified into subclasses, including α-helical, β-sheet, and extended/random-coil peptides (Mahlapuu et al., 2016; Falanga and Galdiero, 2017). The first subclass, α-helical AMPs, are unstructured in aqueous environments but become amphipathic α-helical structures upon contact with biological membranes (Takahashi et al., 2010). Their main activity involves the disruption of bacterial membranes, with a broad antimicrobial spectrum including Gram-positive and Gram-negative bacteria, fungi, and parasites (Lee et al., 2013a; Kim et al., 2014; Raja et al., 2017). In contrast to α-helical peptides, β-sheet AMPs are structured in aqueous solution and do not undergo conformational changes when they interact with membranes (Lee et al., 2016). They also contain cysteine residues that form disulfide bridges, which reinforce their structure and diminish protease degradation (Lee et al., 2016). These AMPs disrupt membranes in a wide range of organisms and have become potential therapeutics as antibacterial, antiviral, antifungal, and anti-inflammatory agents (Panteleev et al., 2015). The third subclass of AMPs, extended/random-coil peptides, lack secondary structures and contain specific amino acids such as histidine (salivary histatins), proline (insect-derived pyrrhocoricin, drosocin, and apidaecin), tryptophan, and arginine (bovine lactoferrin and human lysozyme; Nguyen et al., 2011; Mahlapuu et al., 2016). These AMPs exert their antimicrobial activity, including bactericidal, fungicidal, or antiparasitic effects, through inducing membrane leakage or disturbing nucleic acid synthesis, protein production, or cell-wall synthesis by interacting with intracellular targets (Nguyen et al., 2011; Lombardi et al., 2019). The amino acid sequences of some representatives of each subclass are displayed in Figure 2.

### Cathelicidin LL-37

LL37, first identified as human CAP18, is a human AMP that has a linear cationic α-helical structure (Larrick et al., 1995; Vandamme et al., 2012). It has antibacterial, antifungal, and antiviral activities, promotes angiogenesis and wound healing, and mediates immunomodulatory and inflammatory responses (Bandurska et al., 2015). LL-37 was identified at various sites, including leukocytes (monocytes, neutrophils, T cells, NK cells, and B cells), epithelial cells of the testis, gastrointestinal tract, respiratory tract, and skin (Dürr et al., 2006). LL-37 can act on both Gram-positive and Gram-negative bacteria. Previous studies have reported that LL-37 has antimicrobial activity against various bacterial species: *Escherichia coli* (Smeianov et al., 2000; Aghazadeh et al., 2019), *Klebsiella pneumoniae* (Smeianov et al., 2000), *Pseudomonas aeruginosa* (Smeianov et al., 2000; Travis et al., 2000), *Neisseria gonorrhoeae* (Bergman et al., 2005), and *S. aureus* (Travis et al., 2000; Midorikawa et al., 2003). LL-37 exerts antibacterial activity through disruption of cell membranes and inhibition of cell wall, nucleic acid, or protein synthesis (Zanetti, 2004; Brogden, 2005). In addition, LL-37 was shown to have greater biofilm eradication capacity against *S. aureus* than conventional antibiotics such as gentamicin, vancomycin, rifampin, doxycycline, and cefazolin (Noore et al., 2013; Kang et al., 2019). LL-37 was able to kill *S. aureus* at nanomolar concentrations, while doxycycline and cefazoline acted at millimolar concentrations (Noore et al., 2013). LL-37 also inhibits the initial attachment and biofilm formation of *S. epidermidis* at low concentrations (Hell et al., 2010).

However, MRSA tends to have elevated resistance to LL-37 compared to MSSA (Ouhara et al., 2008). Aureolysin, a metalloproteinase produced by some *S. aureus* strains, was shown to cleave and inactivate LL-37—the more aureolysin produced by an *S. aureus* strain, the less susceptibility of this strain to the antimicrobial fragment LL-17-37 (Sieprawska-Lupa et al., 2004). LL-37 derivatives have been investigated to improve the quality of LL-37 in terms of their stability, hemolysis and cytotoxicity, cell selectivity, and biofilm eradication (Chen et al., 2021; Ridyard and Overhage, 2021). D-LL-37, an LL-37 derivative in which all amino acids are changed to the D-form, displayed protease-resistance properties while possessing biofilm-inhibition capacity equal to the L-peptide isomer LL-37 (inhibiting ~40% biofilm formation at a concentration of 10 μg/ml) and immunostimulatory activity and wound-healing properties on the host higher than the L-peptide (Dean et al., 2011). SK-24, which corresponds to residues 9–32 of LL-37, demonstrated killing activity against *S. aureus* and *Staphylococcal* biofilm reduction, which was superior to LL-37 and several other derivatives (Zhang et al., 2021). KE-18, a derivative corresponding to residues 15–32 of LL-37, showed significant biofilm prevention against *S. aureus* (Luo et al., 2017). KR-12-a5 is another LL-37-derived peptide, a KR-12 analogue corresponding to residues 18–29, which exhibits higher antimicrobial activities against MRSA than LL-37 (Kim et al., 2017).

### Defensins

Defensins are AMPs that belong to the β-sheet subclass and carry six disulfide-linked cysteine residues and 4–10 arginine residues per molecule (Ganz et al., 1985). Defensins have been widely discovered from plants, insects, and mammals, with broad antimicrobial activity against Gram-positive and Gram-negative bacteria, enveloped- and non-enveloped viruses, and yeast and filamentous-phase fungi (Ganz et al., 1990; Gao et al., 2021). Various functions of defensins have been investigated, including pore formation, neutralization or inactivation of secreted toxins, modulation of the immune system and enhancement of antibacterial effects, and induction of cytokine and chemokine expression to fight against bacteria (Gao et al., 2021).

Two main defensin subfamilies, α- and β-defensins, have been reported in humans and some other mammals (Ganz, 2003), while another subfamily, θ-defensins, was later identified in non-human primates such as rhesus macaque monkeys or baboons (Tang et al., 1999; Garcia et al., 2008). Alpha-defensins differ from beta-defensins by the length of peptide chains between the six cysteine residues and the connecting patterns of the cysteine pairs to form disulfide bonds (Ganz, 2003). Human α-defensins have been identified from human neutrophils, gastrointestinal tract, and epithelial tissues, and β-defensins have been identified from neutrophils, leukocytes, epithelial cells, blood plasma, and urine (Schneider et al., 2005; Gao et al., 2021). To date, six human α-defensins have been reported, including human neutrophil peptides 1–4 (HNPs1–4) and human enteric defensins 5–6 (HD5 and HD6; Gao et al., 2021). Among HNPs1–4, HNP2 demonstrated powerful antibacterial activity against *S. aureus*, surpassing the other three HNPs (Ericksen et al., 2005). HD5 and HNP2 exhibited comparable activity against *S. aureus*, while HD6 did not exhibit antibacterial activity (Ericksen et al., 2005).

More than 30 human β-defensin genes have been discovered; however, only a few have been intensively investigated (Schutte et al., 2002; Vankeerberghen et al., 2005). They were identified in various organs and solutions inside the human body, for example, in the hemodialysis solution from patients with renal failure, gastrointestinal tract, urogenital tract, respiratory tract, oral cavity, oral epithelium, damaged psoriasis skin, skin, tonsil, testicles, and antrum (Gao et al., 2021). Among the four primarily studied human β-defensins (hBD-1–4), hBD-3 exhibited antibacterial activity against *S. pyogenes* and *S. aureus*, including multidrug-resistant *S. aureus*, through the cell wall perforation effect (Harder et al., 2001; Schneider et al., 2005). Additionally, hBD-3 has been reported to effectively eliminate staphylococci biofilms, even with MRSA and MRSE, and was significantly more effective than clindamycin (Huang et al., 2012; Lee et al., 2013b). At low concentrations (4–8 μg/ml), hBD-3 effectively restricted bacterial adhesion after 6 h and biofilm formation after 12 h against MRSA and MRSE (Zhu et al., 2013). However, MRSA was reported to have higher resistance to hBD-3 than MSSA, with 55% of the tested MRSA strains exhibiting greater than 20% survival under treatment with 1 μg/ml hBD-3 compared to 13% of the tested MSSA strains (Midorikawa et al., 2003). Nevertheless, combinations of defensins with methicillin or β-defensins and CAP18 can have a synergistic effect on *S. aureus*, including MRSA (Midorikawa et al., 2003). H4, a chimeric human defensin that combines the sequences of hBD-3 and hBD-4, showed superior antibacterial activity against *S. aureus* compared with that of hBD-3 and hBD-4 and conferred high salt tolerance (Yu et al., 2021). hBTD-1 and [D]hBTD-1, chimeric analogues of human β-defensin 1 and θ-defensin, respectively, exhibited considerable activity against *S. aureus* biofilms as well as planktonic forms (Mathew et al., 2017).

### Histatins

Histatins are histidine-rich proteins that are secreted by human parotid and submandibular glands into the salivary glands (Oppenheim et al., 1988). They were shown to have a broad spectrum of antibacterial and antifungal activities (Tsai and Bobek, 1998; Rothstein et al., 2001; Sajjan et al., 2001). Histatin 5 (Hst5) was shown to kill 60–70% of *S. aureus* in 10–100 mM sodium phosphate buffer (NaPB) but had limited activity against *S. aureus* biofilms (Du et al., 2017). Hst 5 may attack *S. aureus* through multiple targets and energy-independent mechanisms (Du et al., 2017).

P-113, a histatin derivative in which residues 4–15 are the same as those of histatin 5, showed a high bactericidal effect on MRSA (Sajjan et al., 2001; Giacometti et al., 2005). Compared to histatin 5, several synthetic histatin analogues, e.g., dhvar 1 and dhvar2, demonstrated increased antibacterial activity against MRSA (Helmerhorst et al., 1997). At a concentration of 2 mg/ml, dhvar1, dhvar4, and dhvar5 exhibited an antibacterial effect against *S. epidermidis* (Elving et al., 2000).

## Bacterial AMPs That Have Antibacterial Activity Against Staphylococcal Pathogens

### Classification of Bacterial AMPs

**Figure 3:**
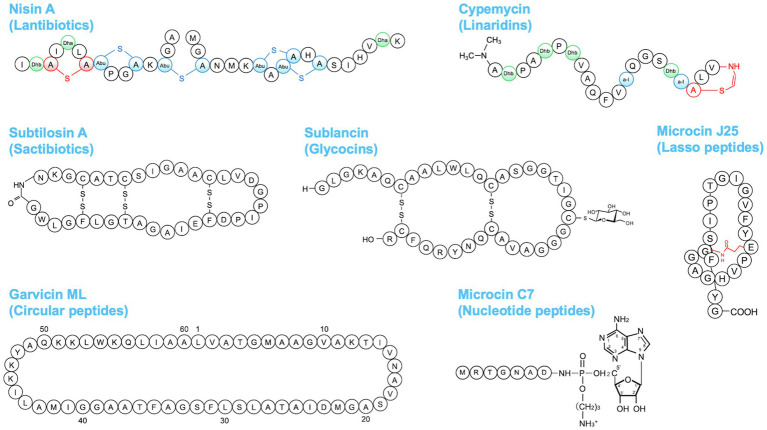
Amino acid structures of some bacteriocins belonging to different groups.

Bacteriocins are ribosomally synthesized AMPs produced by bacteria and have been classified based on the producer organism, inhibitory spectrum, molecular size, chemical structure, mode of action, or plasmid nature (Jack et al., 1995; Ennahar et al., 2000; Oscáriz and Pisabarro, 2001; Kumariya et al., 2019). Various classification schemes for bacteriocins have been proposed over the years. In this review, we adopt an updated classification by Soltani et al. (2021). The classification of bacteriocins is presented in Table 1. The bacteriocins from Gram-positive and Gram-negative bacteria were classified into two classes, namely class I and class II. Class I bacteriocins are ribosomally synthesized and posttranslationally modified peptides (RiPPs; Arnison et al., 2013) with molecular masses <5 kDa. Class I bacteriocins are further subdivided based on their modifications. They can be divided into lantibiotics, sactibiotics, linaridins, thiopeptides, glycocins, circular peptides, and bottromycins from Gram-positive bacteria; nucleotide peptides and siderophore peptides from Gram-negative bacteria; linear azol(in)e-containing peptides (LAPs) and lasso peptides from both Gram-positive and Gram-negative bacteria; and cyanobactins produced by cyanobacteria. Among these, lantibiotics have been widely investigated for therapeutic applications (Soltani et al., 2021; Fernandes and Jobby, 2022). Lantibiotics usually contain 19–38 amino acids that carry unusual amino acid residues, namely lanthionine, β-methyllanthionine, and dehydrated amino acids (Willey and van der Donk, 2007; Arnison et al., 2013). Lanthionine and β-methyllanthionine are conducted from dehydration of serine and threonine, yielding di-dehydroalanine and di-dehydrobutyrine residues, respectively, followed by forming thioether linkages for stabilization. Other class I-bacteriocins have their characteristic modification, such as sactibiotics with sulfur to α-carbon linkage, glycocins with glucosylated cysteine, thiopeptides with a 6-membered nitrogen-containing ring, bottromycins with methylated amino acids, and C-terminal decarboxylated thiazole (Table 1; Figure 3). Class II bacteriocins consist of unmodified peptides, which are further divided into three subclasses: pediocin-like single peptides, unmodified single peptides, and two-peptide bacteriocins.

**Table 1:** Classification of bacteriocins from Gram-positive and Gram-negative bacteria.

Class	Group	Characteristics	Examples of bacteriocins (producer strain)
Class I (posttranslationally modified bacteriocins - RiPPs)	Lantibiotics	Contain lanthionine and 3-methyl-lanthionine residues Some contain two lantibiotic peptides	Nisin (*Lactococcus lactis*) Epidermin (*Staphylococcus epidermidis*) Nukacin (*Staphylococcal* sp.) Mutacin I, II, III/1140, IIIb/B-Ny266 (*Streptococcus mutans*) Mutacin Smb, K8 (*Streptococcus mutans*) Lacticin 3147 (*Lactococcus lactis*) Haloduracin (*Bacillus halodurans*)
	Sactibiotics	Contain sulfur to α-carbon linkage(s)	Subtilosin A (*Bacillus subtilis*) Thuricin CD (*Bacillus thuringiensis*) Ruminococcin C (*Ruminococus gnavus*)
	Linaridins	Linear peptides Contain dehydroamino acids, allo-isoleucine, N-terminal N,N-dimethyl-alanine and C-terminal 2-aminovinyl-D-cysteine (Avi-Cys)	Cypemycin (*Streptomyces* sp.)
	Thiopeptides	Macrocyclic peptides Contain a characteristic six-membered nitrogen-containing ring, oxazole/thiazol(in)e rings and/or dehydroamino acids	Thiostrepton (*Streptomyces azureus*)
	Glycocins	Contain S-glucosylated cysteine(s)	Sublancin (*Bacillus subtilis* 168)
	Circular peptides	N-to-C cyclized unmodified single peptides	Enterocin AS-48 (*Enterococcus faecalis*) Garvicin (*Lactococcus garvieae*) Gassericin A (*Lactobacillus gasseri*)
	Bottromycins	Macrocyclic peptides with a linear tail Contain an amidine moiety, methylated amino acids and a C-terminal decarboxylated thiazole	Bottromycin A2 (*Streptomyces bottropensis*)
	Nucleotide peptides	Contain a nucleotide part	Microcin C (*Escherichia coli*)
	Siderophore peptides	Contain a non-ribosomal siderophore-type modification anchored at a serine-rich C-terminal region	Microcin E492 (*Klebsiella pneumoniae*) Microcins H47, M (*Escherichia coli*)
	Linear azol(in)e- containing peptides (LAPs)	Linear peptides containing combinations of thiazole and oxazole heterocycles	Microcin B17 (*Escherichia coli*) Listeriolysin S (*Listeria monocytogenes*)
	Lasso peptides	Contain only unmodified amino acids Characterized by an entangled [1]rotaxane topology (lasso fold)	Microcin J25 (*Escherichia coli*)
	Cyanobactins	Macrocyclic peptides Contain azol(in)e heterocycles and D-stereocentres Some contain a prenylated amino acid	Patellamide A (*Prochloron* spp.) Sphaerocyclamide (*Sphaerospermopsis* sp.)
Class II (unmodified bacteriocins)	Pediocin-like single peptides	Contain YGNGV-motif	Pediocin PA-1 (*Pediococcus acidilactici*) Enterocin CRL35 (*Enterococcus mundtii*) Carnobacteriocin BM1 (*Carnobacterium piscicola*)
	Unmodified single peptides	Non-YGNGV-motif linear single peptides	Epidermicin NI01 (*Staphylococcus epidermidis*) Lactococcin A (*Streptococcus cremoris*) Microcin V (*Escherichia coli*) Microcin L (*Escherichia coli*) Microcin S (*Escherichia coli*)
	Two-peptides	Two or more unmodified peptides	Mutacin IV, V, VI, N (*Streptococcus mutans*) Plantaricin F (*Lactobacillus plantarum*) Lactacin F (*Lactobacillus johnsonii*)

### Nisins

Nisin is a 34-amino acid lantibiotic produced by Gram-positive bacteria, including *Lactococcus*, *Staphylococcus*, and *Streptococcus* species (Gross and Morell, 1971; O’Connor et al., 2015; O’Sullivan et al., 2020; Figure 3). To date, several variants of nisin have been reported, for example, nisin A (Gross and Morell, 1971), nisin Z (Mulders et al., 1991), nisin Q (Zendo et al., 2003), nisin U (Wirawan et al., 2006), nisin F (de Kwaadsteniet et al., 2008), nisin H (O’Connor et al., 2015), nisin O (Hatziioanou et al., 2017), nisin J (O’Sullivan et al., 2020), and nisin P (Garcia-Gutierrez et al., 2020). Nisin has an antibacterial effect against a wide spectrum of Gram-positive bacteria, including staphylococci, streptococci, enterococci, bacilli, and listeria. Nisin binds to lipid II, which is a membrane component required for peptidoglycan biosynthesis, and then permeabilizes the cell membrane and inhibits cell wall synthesis (Breukink and de Kruijff, 1999; Lubelski et al., 2008). Additionally, nisin was shown to cause cell shrinkage and chromosomal DNA condensation in a MRSA model, suggesting that nisin interferes with DNA replication or segregation in the bacteria (Jensen et al., 2020).

Nisin A exhibited high antibacterial activity against both planktonic and biofilm *S. aureus* cells (Okuda et al., 2013). The combination of nisin A and vancomycin was reported to effectively inhibit *S. aureus* biofilm formation and reduce the thickness of preformed biofilms produced by multidrug-resistant *S. aureus* (Angelopoulou et al., 2020). Nisin A and nisin Z differ by a single substitution at the 27th amino acid residue, with a histidine in nisin A and an asparagine in nisin Z. The structural alteration gives nisin Z a higher solubility and diffusion ability while maintaining its antimicrobial activity compared to nisin A, providing an advantage for nisin Z in the food preservation industry (de Vos et al., 1993; Laridi et al., 2003). The combination of nisin Z (1 μg/ml) and methicillin (32 μg/ml) significantly reduced the growth of MRSA (3.1 log reduction after 3 h of treatment; Ellis et al., 2019). Nisin U, a variant produced by *Streptococcus uberis*, exhibited an inhibitory effect against some staphylococci, such as *Staphylococcus simulans* and *Staphylococcus cohnii*, but not against *S. aureus* (Wirawan et al., 2006). Nisin F is a variant produced by *L. lactis* subsp. *lactis* and showed antimicrobial activity against *Staphylococcus carnosus* and *S. aureus* (de Kwaadsteniet et al., 2008). Nisin J, a nisin variant produced by *S. capitis*, demonstrated high antimicrobial activity against *Staphylococcus* species, including MRSA (O’Sullivan et al., 2020). Nisin H, a bacteriocin produced by *Streptococcus hyointestinalis* and conferring an intermediate structure between lactococcal nisin A and streptococcal nisin U, also exhibited a bactericidal effect against *S. aureus* (O’Connor et al., 2015). Nisin P was produced by *Streptococcus agalactiae* and showed antibacterial activity against staphylococci, but the effect was not as high as nisin A and H (Garcia-Gutierrez et al., 2020). Ripcin B–G, a synthetic peptide generated by the fusion of ripcin and the C-terminal end of nisin (1–20), exhibited stronger and selective bactericidal activity against *S. aureus*, including MRSA (Zhao and Kuipers, 2021). Nisin encapsulated in nanofibers made of polyvinyl alcohol, wheat gluten, and zirconia exhibited well-controlled release and high inhibition activity against *S. aureus* (H. Wang et al., 2015). Nisin-biogel, a delivery system for nisin based on guar gum gel, has been developed and displayed antimicrobial activity against *S. aureus* (Jesus et al., 2021). At subinhibitory levels, it suppressed some virulence factors, such as the factors related to biofilm formation, coagulase, and protein A; however, the expression of some other virulence genes, such as *spA* (staphylococcal protein A), *coa* (coagulase), *icaA* (intracellular adhesin A), and *icaD* (intracellular adhesin D), was elevated, requiring a thorough consideration of the optimal dosage when applying nisin in clinical practice (Jesus et al., 2021).

### Epidermins

**Figure 4:**
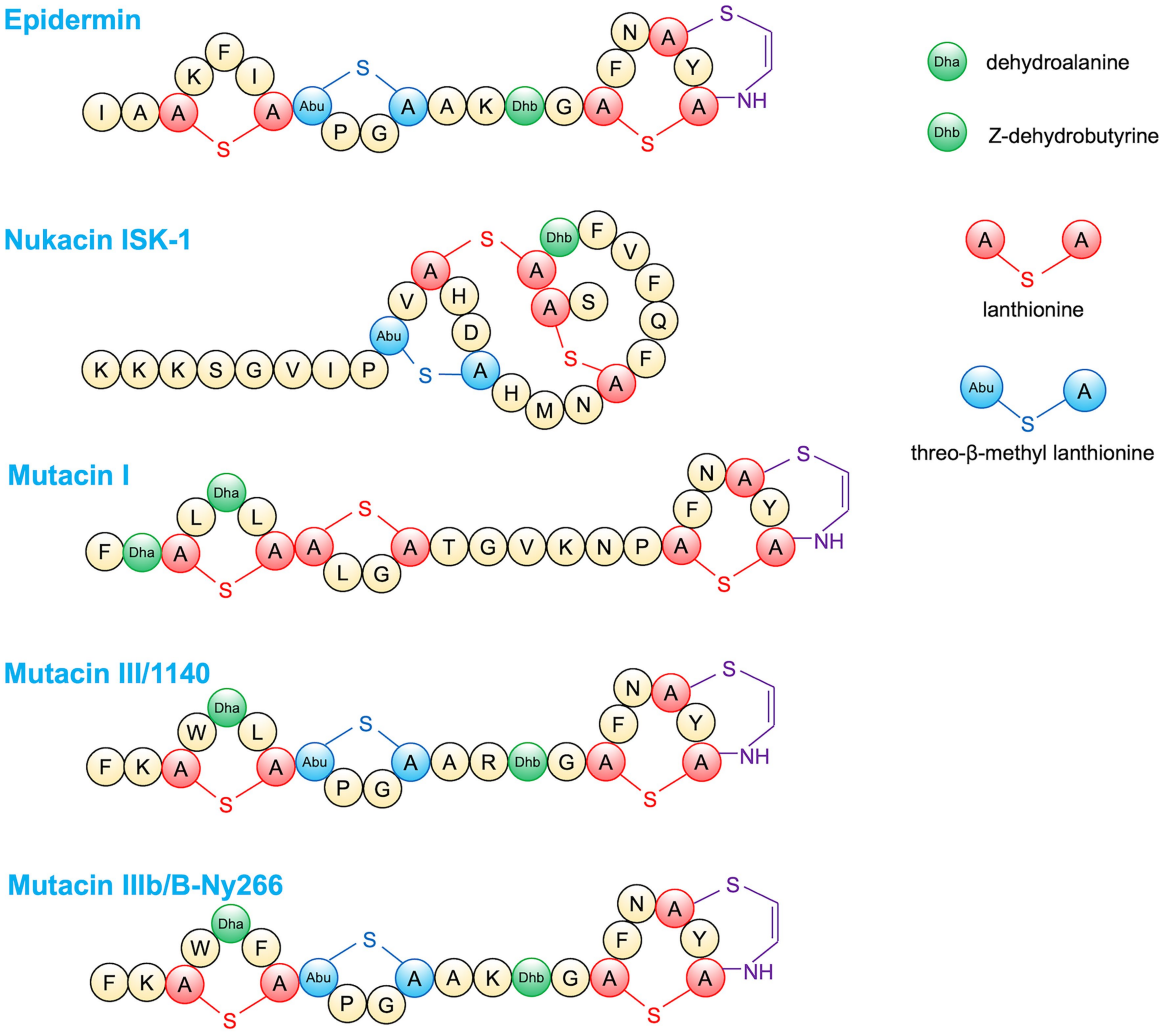
Amino acid structures of lantibiotic bacteriocins.

Epidermin is a 21-amino acid lantibiotic produced by *S. epidermidis* that exhibits an antimicrobial effect against staphylococci and streptococci (Schnell et al., 1988; Figure 4). Epidermin kills bacteria by inhibiting cell wall synthesis by interacting with the cell wall precursor lipid II and sometimes by causing pore formation (Bonelli et al., 2006).

Epidermin significantly reduced the *S. epidermidis* cells attached to silicone catheters in an *in vitro* catheter colonization model (Fontana et al., 2006). Epidermin exhibited antibacterial activity against >85% of tested *S. aureus* (165 strains) involved in bovine mastitis (Varella Coelho et al., 2007). In another study, epidermin showed antibacterial activity against 81.3% of tested *S. aureus* involved in human infections, including MRSA endemic clones in Brazil (Nascimento et al., 2006). Epidermin also exhibited antibacterial effects against *S. haemolyticus*, *S. capitis*, *S. simulans*, *S. saprophyticus*, *S. hominis*, and *S. epidermidis*, although no activity was observed against some tested *S. aureus* (Nakazono et al., 2022).

### Nukacins

Nukacin is a type A(II) lantibiotic that was first identified in 1998 from *S. warneri* ISK-1 (Kimura et al., 1998). This peptide consists of 27 amino acid residues and arrests cell wall biosynthesis by binding to lipid II (Islam et al., 2012; Figure 4). Nukacin ISK-1 showed a bacteriostatic effect against *Bacillus subtilis* by stopping cell growth without pore formation (Asaduzzaman et al., 2009) while exhibiting bactericidal activity against *Micrococcus luteus* and *S. simulans via* pore formation and cell lysis (Roy et al., 2014). Several variants of nukacin have been reported, including nukacin KQU-131 produced by *S. hominis* (Wilaipun et al., 2008), nukacin 3299 produced by *S. simulans* (Ceotto et al., 2010), and nukacin IVK45 and nukacin KSE650 produced by *S. epidermidis* (Janek et al., 2016; Nakazono et al., 2022). Nukacin ISK-1 exerted a bacteriostatic effect against MRSA planktonic cells; however, activity against biofilm cells was not observed (Okuda et al., 2013). Nukacin 3299 exhibited antibacterial activity against 66.7% (18/27) of *S. aureus* strains involved in bovine mastitis (Ceotto et al., 2010). Nukacin KSE650 showed antibacterial activity against *S. haemolyticus*, *S. capitis*, *S. simulans*, *S. saprophyticus*, *S. hominis*, and *S. epidermidis*, although no activity was observed against some tested *S. aureus,* while Nukacin ISK-1 showed an antibacterial effect against *S. aureus* (Nakazono et al., 2022). The difference of four amino acid residues between Nukacin ISK1 and Nukacin KSE650 mature peptide caused different susceptibility against *S. aureus* strains.

### Mutacins

Mutacins are bacteriocins produced by *Streptococcus mutans*, which have been classified into two types: lantibiotics containing unusual amino acid residues and non-lantibiotics consisting of unmodified peptides (Merritt and Qi, 2012). The lantibiotic mutacins include mutacin I, II, III/1140, B-Ny266 (Figure 4), Smb, and K8 and usually confer a wider spectrum of activity. In contrast, non-lantibiotics, such as mutacin IV, V, VI, and N, play an important role at the *S. mutans* intraspecies level and in closely related species (Merritt and Qi, 2012). Lantibiotic mutacins were reported to have bactericidal activity through pore formation and inhibiting cell wall synthesis (Hasper et al., 2006). Against *S. aureus*, mutacin III exhibited the highest antibacterial activity; mutacin I, IIIb (mutacin B-Ny266), and IVb showed intermediate activity, while mutacin II, IV, K8, and Smb had almost no effect (Watanabe et al., 2021).

### Other Bacterial AMPs

Garvicin KS is a leaderless multipeptide bacteriocin produced by *Lactococcus garvieae* (Thapa et al., 2020). Garvicin KS showed bactericidal activity against 50/53 tested *S. aureus* strains (Chi and Holo, 2018). Against the least sensitive strain, the combination of nisin and garvicin KS showed a synergetic effect by completely killing the bacteria after 12 h. The combination of farnesol and garvicin KS was not effective; however, combinations of farnesol and nisin or the three compounds rapidly eradicated *S. aureus* (Chi and Holo, 2018). Garvicin KS and micrococcin P1 displayed a synergetic effect against *S. aureus* biofilms, including MRSA (Kranjec et al., 2020). Pep5 is a 34-amino acid residue lantibiotic produced by *S. epidermidis* (Kaletta et al., 1989). Pep5 exhibited inhibitory activity against 77.2 and 87.5% of the tested coagulase-negative staphylococci and *S. aureus*, respectively (Nascimento et al., 2006).

## Effect of AMPs on Staphylococcal Infection Models and Clinical Trials Using AMPs

To evaluate the effect of AMPs including human AMPs and bacteriocins on *S. aureus* and the host, *in vivo* experiments using *S. aureus*-infected animal models were conducted. In a skin infection murine model, garvicin KS and micrococcin P1 displayed synergistic effects against MRSA (Ovchinnikov et al., 2020). In a rat *S. aureus*-induced uterine endometritis model, nisin (25 mg/kg) administration significantly restored the inflammation of the endometrium and improved the expression of several serum cytokines, which showed high expression in the endometritis (Jia et al., 2019). Nisin-eluting nanofibers were also shown to be effective against skin infection by MRSA in mice (Heunis et al., 2013). Mersacidin, a lantibiotic produced by *Bacillus* sp., was shown to eradicate the colonization of human-derived MRSA in a mouse rhinitis model (Kruszewska et al., 2004). Sublancin, a glycosylated AMP produced by *B. subtilis*, exhibited antibacterial and immunomodulatory effects against a MRSA-infected mouse model that induced intraperitoneal (Wang et al., 2017; Li et al., 2021) or intestinal injury (Wang et al., 2014). K2A and R13A, two analogues of mutacin 1,140, have been reported to improve pharmacokinetics *in vivo* and efficiently rescue mice infected with MRSA at 10 mg/kg (100% protection) or 2.5 mg/kg (50% protection; Geng et al., 2018). In an intraperitoneal infection mouse model, mutacin B-Ny266 effectively protected the mice from mortality induced by *S. aureus.* At a dose <1 mg/kg, mutacin B-Ny266 showed a comparable ED_50_ (effective dose protecting 50% of the animals) to that of vancomycin (Mota-Meira et al., 2005). Epidermicin NI01, a synthetic AMP, effectively protected *Galleria mellonella* larvae from MRSA infection without signs of toxicity or stimulating the host immune system (Gibreel and Upton, 2013).

Regarding human AMPs, many experiments to show the effect of AMPs, especially LL-37 and beta-defensin, on various *S. aureus* infection animal model have been reported. The application of LL-37 promoted tissue regeneration including re-epithelialization and angiogenesis in MRSA-infected surgical wounds (Simonetti et al., 2021). Hybrid peptide CaD23 consisted of LL-37 and human beta-defensin-2 showed a good efficiency with the reduction of *S. aureus* cells by 94% in a murine keratitis model (Ting et al., 2021). The LL 37 derivatives, 17tF-W, eliminated MRSA USA300 cells in catheter and its surrounding tissues of a murine infection model (Narayana et al., 2019). Both LL-37 and IDR-1 (an innate defense regulator peptide) exhibited immunomodulation effects and restored pulmonary function in mice with MRSA pneumonia (Sun et al., 2013). Histatin 5, Dh5 (residues 11–24 of histatin), P-113, Dhvar4 (an increased amphipathicity variant from Dh5), and Dhvar5 (a reduced amphipathicity variant from Dh5) showed bactericidal effects against *S. aureus*, including MRSA, *in vivo* (Welling et al., 2007). In an *in vivo* MRSA osteomyelitis prevention model, 24 mg Dhvar-5 beads showed a significant reduction in bacterial load inoculated in rabbit femora compared to the control; however, complete sterilization of the femora could not be observed (Faber et al., 2004). ^99m^Tc-HBD-3, a human β-defensin 3 radiolabeled with ^99m^Tc, demonstrated favorable uptake of AMPs at the infected site in an *S. aureus-*infected rat model (Follacchio et al., 2019). HDMP, a human defensin-6 mimic peptide, significantly rescued mice with MRSA bacteremia at a survival rate as high as 100%, which was higher than that of vancomycin (83.3%) at the same dosage (5 mg/kg; Fan et al., 2020).

The promising results of AMP activity observed in preclinical studies have led to investigations in human clinical trials to evaluate their safety and effectiveness. Clinical trials targeting *S. aureus* infections have been carried out (Table 2). Compared to placebo, topical LL-37 treatment significantly improved the healing rate of hard-to-heal venous leg ulcers, without any local or systemic safety concerns (Grönberg et al., 2014). Intranasal treatment with LTX-109, a chemically synthesized, peptide-mimetic drug, was reported to effectively eradicate the persistent colonization of MRSA and MSSA in the nasal cavity without the signs of adverse effects (Nilsson et al., 2015). PLG0206, an engineered AMP, efficaciously reduced the bacterial count, including *S. epidermidis* and *S. aureus,* in patients with chronic periprosthetic joint infections (Huang et al., 2021). GSK132232, a synthetic AMP, rapidly decreased lesion size and pain in acute bacterial skin and skin structure infections, although several mild-to-moderate adverse effects were observed, including nausea, vomiting, diarrhea, and headache (Corey et al., 2014). Numerous AMPs have undergone clinical trials; however, only a few AMPs are currently approved for clinical application, including nisin, gramicidin, polymyxins, daptomycin, and melittin (Dijksteel et al., 2021).

**Table 2:** Some antimicrobial peptides (AMPs) under investigation and clinical phase of development for treatment of *Staphylococcal* infections.

Peptide name	Description	Target	Administration	Phase	Clinical trial ID	Mechanism	References
Nisin	Polycyclic lantibiotic	Ventilator Associated Pneumonia	Oral		NCT02928042	Depolization of cell membrane	
LL-37	Human cathelicidin	Hard-to-heal venous leg ulcers	Topical polyvinyl alcohol viscous-based solution	I/II	EU Clinical Trials 2012-002100-41	Membrane disruption Immunomodulation	Grönberg et al., 2014
OP-145	Derivative of LL-37	Chronic suppurative otitis media	Ear drops	I/II	ISRCTN84220089	Membrane disruption Immunomodulation	
PMX-30063	Defensin mimetic	Acute Bacterial Skin Infections Caused by *Staphylococcus aureus* (MSSA)	Intravenous	II	NCT01211470	Membrane disruption Immunomodulation	
LTX-109	Synthetic tripeptide	Persistent nasal *S. aureus* carriers (MRSA/MSSA) Non-bullous Impetigo	Topical hydrogel	I/II	NCT01158235 NCT01803035	Membrane disruption	Nilsson et al., 2015
XF-73 (Exeporfinium Chloride)	Derivative of porphyrin	Commensal *S. aureus* nasal carriage	Topical nasal gel	II	NCT03915470	Membrane disruption	
PLG0206	Engineered cationic antimicrobial peptide	Periprosthetic Joint Infection	Intravenous	I	NCT05137314	Membrane disruption	Huang et al., 2021
Friulimicin B	Cyclic lipopeptide	Community Acquired Pneumonia Staphylococcal Skin Infections	Intravenous	I	NCT00492271	Membrane disruption	
Omiganan	Derivative of Indolicidin	Catheter Infections/Colonization in Patients With Central Venous Catheters Topical Skin Antisepsis	Topical gel	III	NCT00231153 NCT00608959	Membrane disruption Immunomodulation	
DPK-060	Derivative of Kininogen	Acute External Otitis	Ear drops	II	NCT01447017	Membrane disruption Immunomodulation	
GSK1322322	Synthetic hydrazide	Acute Bacterial Skin and Skin Structure Infection	Oral	II	NCT01209078	Peptide deformylase inhibitor	Corey et al., 2014

## Emergence of Resistance to AMPs

**Figure 5:**
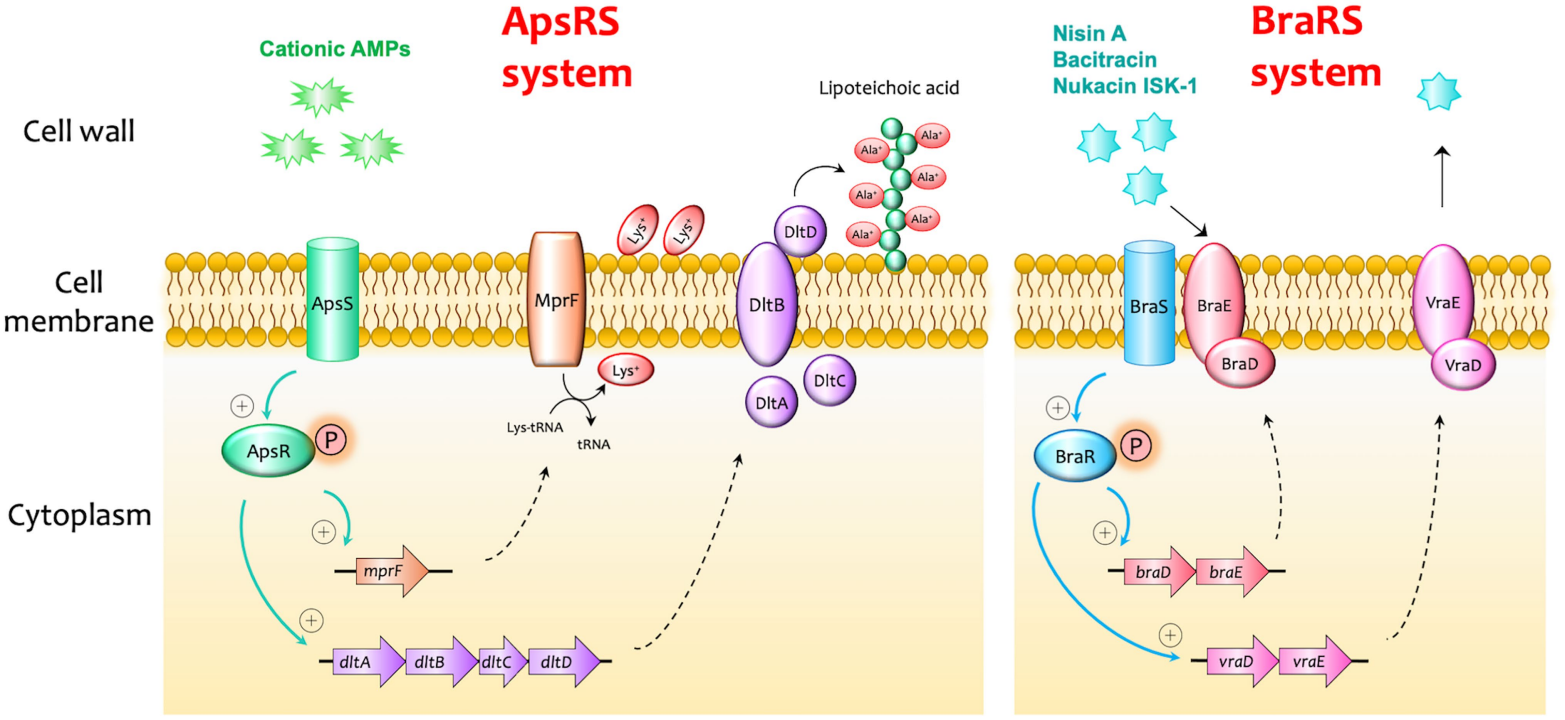
Examples of bacteriocin resistance mechanisms in *Staphylococcus aureus*. I. In the ApsRS system, the sensing of cationic antimicrobial peptides (AMPs) results in the autophosphorylation of ApsS, followed by the phosphorylation of ApsR. The phosphorylated ApsR can bind to the upstream regions of *mprF* and *dltABCD*, increasing the expression of these factors. MprF is associated with the addition of lysine to phosphatidylglycerol in the cell membrane, and DltABCD is associated with the addition of alanine to teichoic acids on the cell wall. Amino acid incorporation causes a shift to a weak negative charge on the cell surfaces and makes the cell less sensitive to cationic AMPs. II. In the BraRS (NsaRS)/BraDE system, sensing of nisin A by BraDE results in the autophosphorylation of BraS, followed by the phosphorylation of BraR. The phosphorylated BraR can bind to the upstream region of *vraDE*, giving rise to the increased expression of an ABC transporter VraDE which expels the AMPs from the cell.

**Figure 6:**
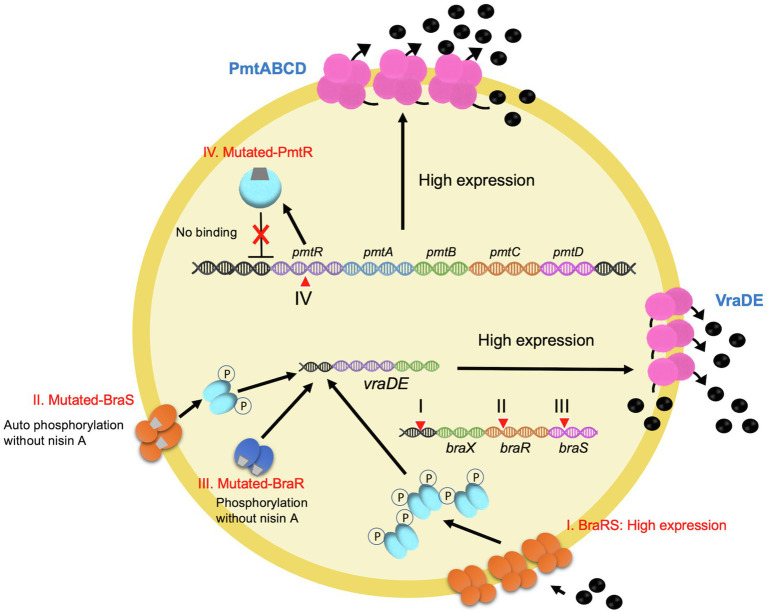
Schematic diagram of the nisin A highly resistant mechanism. I. A point mutation in the promoter region results in the higher expression of *braXRS*. This leads to the increased induction of *vraDE* expression by nisin A. II. A point mutation in *braS* (encoding a sensor protein) causes nisin A-independent phosphorylation of BraS, resulting in increased phosphorylated BraR, which induces a constant expression of *vraDE*. III. A point mutation in *braR* (encoding a response regulator) results in nisin A-independent activation of *vraDE* expression. IV. A point mutation in *pmtR* encoding a negative regulator PmtR for *pmtABCD* expression. Mutated PmtR, which lacks the DNA binding ability, results in a constant *pmtABCD* expression.

Although AMPs represent potential alternative clinical antimicrobials, continuous exposure to AMPs could lead to the development of resistance in formerly susceptible cells. Bacteria can escape bacteriocins through acquired resistance and innate resistance involved in cell wall synthesis, cytoplasmic membrane synthesis, cell envelope alterations, membrane permeability and specific receptor expression, or energy metabolism and transport (de Freire Bastos et al., 2015; Andersson et al., 2016; Soltani et al., 2021). In *S. aureus,* several factors were reported to be involved in resistance to AMPs (Figures 5, 6). The disruption of either Dlt or MprF results in elevated sensitivity to defensins (Peschel et al., 1999, 2001; Kawada-Matsuo et al., 2013). Dlt is associated with the addition of alanine to teichoic acids on the cell wall, while MprF is associated with the addition of lysine to phosphatidylglycerol in the cell membrane (Figure 5). Amino acid incorporation causes a shift to a weak negative charge on cell surfaces (Peschel et al., 1999, 2001). A transporter of VraDE regulated by one two-component system, BraRS, is associated with resistance to nisin A, Nukacin ISK-1, and bacitracin (Kawada-Matsuo et al., 2013). In BraRS (NsaRS)/BraDE system, sensing of nisin A by BraDE results in the autophosphorylation of BraS, followed by the phosphorylation of BraR (Hiron et al., 2011; Randall et al., 2018). The phosphorylated BraR can bind to the upstream region of *vraDE*, giving rise to the increased expression of *vraDE* (Figure 5). The transporter PmtA-D regulated by PmtR is associated with resistance to beta-defensin 3 and nisin A (Cheung et al., 2018). Another factor, staphylokinase, was also involved in alpha-defensins resistance because staphylokinase directly binds to alpha-defensin, causing the neutralization of its activity (Bokarewa et al., 2006). These factors are generally conserved among *S. aureus* strains. Therefore, *S. aureus* has a natural resistant system against several bacteriocins, although *S. aureus* retained sensitivity when treated with high concentrations of AMPs.

It was questionable whether highly resistance could evolve if exposed to a high concentration of nisin A. After several experimental trials, finally, some research groups were able to obtain highly nisin A-resistant *S. aureus* strains with mutations in BraRS, or PmtR when exposing *S. aureus* to sub-MICs of nisin A (Blake et al., 2011; Arii et al., 2019; Kawada-Matsuo et al., 2020, 2021). Arii et al. (2019) isolated several nisin A highly resistant *S. aureus* strains by exposing sub-MIC of nisin A three times and obtained two types of the mutants, with mutations in *braRS* or *pmtR* (Figure 6). The mutants with *braRS* mutation showed higher expression of VraDE, which is an effector for nisin A resistance, than that of the wildtype, while the mutants with *pmtR* mutation did not show high expression of VraDE. Three mutants with *braRS* mutation had different point mutation sites, including the upstream region of *braXRS*, *braR*, or *braS*. The point mutation upstream of the *braXRS* region was associated with the increased promoter activity, causing high expression of *braRS*. By the point mutation of *braR*, the mutated BraR without phosphorylation was able to bind to the upstream region of *braRS*. The *braS* mutation was found in the histidine kinase region, suggesting that the mutated BraS is autophosphorylated without the stimulation of nisin A. Blake et al. also reported the point mutation of NsaS (BraS) in nisin-resistant *S. aureus* strains. Another nisin A highly resistant *S. aureus* mutant was isolated by the mutation of PmtR with the increased expression of PmtA-D, an ABC transporter, involved in the susceptibility to nisin A and beta-defensin (Kawada-Matsuo et al., 2020, 2021). Since PmtR was a negative regulator for PmtA-D expression, the mutated PmtR could not bind to the upstream region of *pmtA-D*. In addition, this mutant also exhibited increased virulence in a mouse bacteremia model. Dobson et al. (2014) reported that AMP-resistant *S. aureus* strains selected by pexiganan, iseganan, or melittin showed higher survival in *Tenebrio molitor*, an insect model. Therefore, considerations should be taken into account to overcome the emergence of bacteriocin-resistant bacteria. The combination of bacteriocins with different modes of action or the combination of conventional antibiotics and bacteriocins may allow a reduction in dosage and avoid the development of bacteriocin resistance (de Freire Bastos et al., 2015).

## Concluding Remarks and Perspectives

*Staphylococci* including *S. aureus* and CoNS are important human pathogens associated with potentially life-threatening infections. The emergence of drug-resistant *Staphylococci* has significantly challenged the available treatment options, necessitating the discovery of novel therapeutics. AMPs exhibit excellent promise as alternatives to conventional antibiotics due to their broad-spectrum activity; rapid mode of action; low risk of resistance development and anti-inflammatory and immunomodulatory effects; synergistic effects with conventional antibiotics; and clinical efficacy against some multidrug-resistant bacteria. This review discusses potential AMPs, focusing on human AMPs and bacteriocins, which display antibacterial activity against *Staphylococci*, including methicillin-resistant staphylococci, *in vitro* and in some infection models, and presents the current clinical investigation phase of some AMPs. The production costs, cytotoxic effects, reduced efficacy in the body (low stability, high susceptibility to proteolysis, reduced activity in physiological conditions), and resistance development are the major obstacles that challenge the clinical usage of AMPs (Pachón-Ibáñez et al., 2017; Dijksteel et al., 2021). The incorporation of AMPs into artificial materials, the development of innovative formulation or delivery systems, and the combination with conventional antibiotics may provide effective strategies to overcome the disadvantages of AMPs and promote their market authorization as novel AMP-based drugs.

## Author Contributions

ML, MK-M, and HK conceptualized and revised the manuscript. ML drafted the manuscript and tables and produced the figures under the guidance of HK. MK-M acquired the fund for the project. All authors contributed to the article and approved the submitted version.

## Funding

This research was funded by Grant-in-Aid for Scientific Research (C) (grant no: 21K09858) from the Ministry of Education, Culture, Sports, Sciences, and Technology of Japan.

## Conflict of Interest

The authors declare that the research was conducted in the absence of any commercial or financial relationships that could be construed as a potential conflict of interest.

## Publisher’s Note

All claims expressed in this article are solely those of the authors and do not necessarily represent those of their affiliated organizations, or those of the publisher, the editors and the reviewers. Any product that may be evaluated in this article, or claim that may be made by its manufacturer, is not guaranteed or endorsed by the publisher.
